# Vibrant Screens: Remote therapy and counselling through the lens of digital materiality

**DOI:** 10.1177/13634593241234491

**Published:** 2024-02-26

**Authors:** Marjo Kolehmainen

**Affiliations:** University of Turku, Finland

**Keywords:** counselling, COVID-19, mental health, more-than-human care, technology

## Abstract

This article analyses the digital screen as a health technology. In particular, the article asks how screens as a part of therapy settings or counselling practices materialise – or fail to materialise – care. The empirical data comprise interviews with therapy and counselling professionals, whose experiences with technology during the COVID-19 pandemic were my original interest. Adopting a sociomaterial approach to technology use, it scrutinises not only how screens are used, but also how screens themselves act and operate. This approach foregrounds the screen as ‘multiple’, complicating a dichotomous understanding between in-person therapy and remote therapy. The article argues that the screen operates in a variety of ways that might either facilitate or degrade care and is an essential part of more-than-human care in digitalised societies. Acknowledging the agential capacities of all matter, the article also conceptualises screens as ‘vibrant matter’.

## Introduction

In this article, I analyse the digital screen as a health technology. The COVID-19 pandemic caused a rapid transition to remote work using video platforms in several occupational fields (Hargittai, 2022; Williams, 2021), including mental health care. Digital screens proved crucial in maintaining access to mental health care, from rapidly digitalised psychotherapies to online hotlines, from crisis work appointments to peer support platforms. This article chiefly taps into the experiences of Finnish therapy and counselling professionals during the COVID-19 pandemic. Finland saw nationwide restrictions concerning social distancing and recommendations concerning remote work. Also Kela, the state pension office that provides rehabilitation psychotherapy, changed its policy to support video or phone calls instead of in-person therapy sessions. The purpose of rehabilitative psychotherapy is to improve the ability of rehabilitation clients to work and study, and persons aged 16–67 may qualify for compensation from Kela (Kela, n.d). Finnish professionals therefore had to adapt quickly to the ‘new normal’. Screens, from laptop to smartphone, became a central part of therapy settings or counselling practices. Both various popular platforms for video-conferencing (e.g. Zoom, Skype, Teams) and software designed specifically for healthcare or psychotherapy purposes (e.g. video-conferencing tools tailored for medical centres) were utilised. In what follows, I provide a detailed analysis of screens as a health technology. Even if not all the screens in question were originally designed exclusively for mental health care, they nevertheless operate as vital health technologies.

This article brings together insights from various strands of *sociomaterial* research – science and technology studies (STS), vital materialism and the theory of more-than-human care – in an effort to better understand the materiality of technology. Despite the differences in the various strands of this research field, they all foreground a notion of distributed agency that does not privilege one particular activity or actor over another. Subscribing to the view of technologies as actors that to do things within their network or practice (Pols, 2011), I also seek to acknowledge the agency of screens in therapy and counselling sessions. From this vantage point, screens are not passive surfaces to be acted upon only by humans; they actively co-constitute therapy and counselling sessions. Indeed, my approach is indebted to such work on care that has questioned the status of technology as passive and instrumental. As Mol (2008) remarks, within STS, care and technology have been discussed as mutually inclusive. In her pioneering work on telecare, Pols (2012) notes that while technology in care work is often seen as ‘cold’, impersonal and instrumental, this view relies on an understanding of technology as simply passive, inert matter, with only instrumental qualities. This article views digital materiality as a process, not an end product or finished object (Pink et al., 2016: 10). From this point of departure, screens are not only digital surfaces but also come to matter in various open-ended ways. This article seeks to make sense of these matterings – becomings of matter and meaning (Barad, 2012) – to provide novel insights into screens as a health technology.

There is an extensive body of work on health technology in STS, including such studies on webcams that my research can be seen as a continuation to. Pols (2011), for instance, has explored what people looking through webcams do within the rehabilitation of people suffering from severe chronic obstructive pulmonary disease (COPD) or asthma. In her study, she identifies several ways to describe the activities webcams support and perform. In a similar vein, Pols and Willems (2011) have examined the use of webcams in follow-up care from a rehabilitation clinic for people with severe COPD. They argue that when innovative technology such as telecare is put to work, ‘the same’ technology will perform differently. The telekits they explore are associated with two particular goals, either guaranteeing the effectiveness of treatment or providing a window onto the world. These different treatment goals manifested in how the telekit was seen either as a *digital umbilical inhaler* – connecting the patient to the clinic – or as an *inhaler* that supports the patient in getting on with their life (Pols and Willems, 2011: 490). Trondsen et al. (2018) have investigated the use of real-time videoconferencing technology in emergency psychiatry. They identified four contributions of the ‘video-mediated gaze’ to the therapeutic encounter: (1) *immediacy of assessment*; (2) *increased transparency*; (3) *sense of access to the ‘real’ expert*; and (4) *fostering of the patient’s ‘voice’ in therapeutic decisions*. These four aspects have either pragmatic or symbolic value. All these three studies stress how videoconferencing technologies operate in multiple ways.

To complete approaches provided by such STS studies that have focused on the operations on webcams, my article also draws upon vital materialism and work on more-than-human care in order to stress the relational constitution of both screens and humans. The vital materialist approach focuses attention on the living forces that are generated in and through humans’ relationships with nonhumans. From this perspective, other living things as well as non-living matter or things possess agency that can work with – or indeed against – the agency possessed by humans, and together generate new forms of agential capacities (Lupton, 2020). Vital materialist approach builds upon the work by Bennett (2004, 2010), whose concept of ‘thing-power’ emphasises the vitalism that objects possess. This concept provides a fruitful lens through which to recognise the agential capacities of screens. From this perspective, objects such as screens do not possess thing-power on their own, but in relation with other elements of an assemblage, which may include humans (Bennett, 2004; Lupton, 2020). Further, acknowledging the agential capacities of all matter, digital screens included, means also recognising their potential in providing care (see Méndez de la Brena, 2022). Thus, aligning with work on more-than-human care, where care is not seen just as a human matter (Puig de la Bellacasa, 2012, 2017), I take screens to be a part of more-than-human care.

## Background: On screens

This article advances a posthumanist view, where humans are not ontologically separate from technology. Posthuman theory locates the human on a continuum of lively bodies and forces – a continuum that elides conventional dichotomies of life and matter, organic and inorganic, subjective and objective, agency and structure (Bennett, 2020). From this perspective, the human/technology dichotomy requires unpacking as well. Underpinning much research on therapy at a distance, however, is an oppositional model of in-person/remote therapy: such characterisations of teletherapy as, for example, a ‘substitute’ or a ‘simulation’, lead to oversimplified understandings of videoconferencing in mental health care. My approach, locating digital therapy and related technological infrastructures as part of more-than-human worlds, enables a better view of techno-materialities as essential parts of therapeutic processes. From this perspective, then, the COVID-19 pandemic and the related shift away from face-to-face therapy are not predefined as an external disruption to therapy practice, but provide novel networks for the relational co-constitution of therapy and counselling processes. The emphasis is on asking what the digital enables, and what new ways of thinking about the world, engaging with it and being in it, might be pulled into existence or made possible (Sumartojo and Graves, 2021). The screen can thus be viewed as a material object that is part of heterogeneous and dynamic assemblages (see Lupton, 2019).

This article thus contributes to studies of screens that focus on their specific, contextualised uses in health care settings. The screen is at the same time an expressive and perceptible technology (Sobchack, 2004: 135), even if its affordances are not reducible to sight alone. Previous research on digital screens has focused on screens that visualise ready-made content in public places and settings, such as museums or academic settings (e.g. Decuypere and Simons, 2016; Sumartojo and Graves, 2021). In health care settings, screens also play a central role in waiting rooms, where their presence shapes the experience of waiting as an activity (McCarthy, 2001: 198). The content of screening varies from television channels to health-related programming and commercial advertising (McCarthy, 2001: 205). Ready-made content is common, especially in such medical settings where large social, emotional or epidemiological issues are concerned (e.g. pregnancy testing, HIV-testing), since there the screens also serve as outreach tools, targeting particular populations in the hope of behaviour modification (McCarthy, 2001: 203). However, screens also play an increasingly significant role in real-time human-to-human communications such as video calls, even if the particularities of webcams in mental health care have remained largely unexplored. In this article I focus specifically on digital screens that facilitate livestream in mental health care.

Screen issues have also been discussed from the perspective of clinical work. From a psychoanalytic perspective, remote psychoanalytic working touches on the question of the psychic significance and role of physical presence and bounded space in clinical work (Shulman, 2021). A safe holding environment cannot be established in a traditional way in remote therapy, and the therapeutic experience is limited, as the setting is not wholly managed and provided by the analyst alone (Isaacs Russell, 2015: 74). Scholars have identified further problems in studies of remote psychotherapy, with obstacles ranging from distractions to emotional distancing. The assumed dangers have been located in tendencies related to technological difficulties, such as ignoring the significance of disruptions or treating them in terms of technological, rather than psychological, relevance (Isaacs Russell, 2015: 126–127; Shulman, 2021). The screen itself has been seen as causing challenges, as it has been approached from the perspective of inherent separation, not allowing the embodied presence of two human beings in a common space (Isaacs Russell, 2015; Markowitz et al., 2021). Personal relations with screens also matter. It has been argued that some patients have been conditioned to feel a work ethic in front of their computer screen (Markowitz et al., 2021: 242), while others use computers mainly for activities like porn consumption or online shopping (Isaacs Russell, 2015: 131) – all of which has an impact on the therapeutic session. For child patients, remote psychotherapy has been argued to have potentially traumatising effects, since psychic experiences of ‘dislocation’, ‘disembodiment’ and ‘psychic slippage’ might occur in relation to the object ‘on’ or ‘in’ the screen (Shulman, 2021). In short, the associations have been pretty negative.

Nevertheless, from a historical perspective, screen technology is anything but ‘external’ to healthcare, mental care included. In the history of medicine and health care, screening technology, for example, X-ray or ultrasound, has been central (Cartwright, 1995). This long history of bodily analysis and surveillance in medicine and science is critically tied to the history of the development of the cinema as a popular cultural institution and a technological apparatus, with many of the techniques and instruments that contributed to the emergence of the cinema having been designed and used by scientists for optics and physiology (Cartwright, 1995). The visual techniques associated with neurology that prevailed through the period in which psychoanalysis became institutionalised thus deserve consideration as a set of practices complicit with psychoanalysis in the twentieth-century medical management of mental illness (ibid., 48). The overlap between psychoanalysis and screens is also manifested in the fact that psychoanalytical approaches were originally central to cinema studies (Kuhn, 2009). This rich history itself reminds us that humans are not easily separated from screens, but rather become through their relations with screens (see Coleman, 2008).

This article thus departs from seeing humans and screens as separate and separable entities between which relations operate, instead seeing them as constituted through their relationality. It aims to make both empirical and theoretical contributions: (1) Through a detailed empirical investigation, it seeks to produce new knowledge on the ways in which digital screens come to matter in mental health care and (2) attempts to provide novel insights into more-than-human care through a careful consideration of the ways in which technology becomes part of the mental health care. To achieve these aims, the article brings together insights from STS, vital materialism and the theory of more-than-human care, as depicted earlier in this article. There is no prior work on digital screens in mental health care, despite the significance of the digital screen in remote therapy and counselling. Drawing on the view that human subjects are part of and inseparable from more-than-human worlds (Lupton, 2019), it points out that mental health is not only a human matter.

## Data and methodology

This article empirically draws upon interview data. I conducted 41 thematic interviews in which psychologists, psychotherapists, family counsellors, crisis workers, sex therapists and other counselling professionals discussed their experiences with technology during the COVID-19 pandemic. The scope of the professionals is wide, coming with a potential limitation that it might not invite as much interviewees’ reflection about the particularities of different therapy modalities, therapist types or particular groups of clients as a limited focus on certain types of therapy would do. However, rather than generating information from the vantage point of particular therapy modality, my original interest laid in the wide field of therapy and counselling work. Consequently, my aim was to document the interviewees’ experiences concerning the transition to remote consultation in real time, and in the unforeseen pandemic circumstances (see Hargittai, 2022). I recruited interviewees in different ways, including by an open call on social media (e.g. on both my personal Twitter and personal Facebook accounts as well as on the home page of *Intimacy in Data-Driven Culture*, a research consortium funded by The Strategic Research Council at the Academy of Finland [327391]) and contacting several organisations providing counselling services. Altogether I interviewed 39 individuals, self-identified as female (*N* = 30) and male (*N* = 9). Most were aged between 30 and 50 years (*N* = 31), apart from six interviewees aged 55 to 59 years and two aged 25–29 years. The interviews varied in length. Two individuals were interviewed twice, on my initiative, as they had responded in a wordy, detailed and reflective manner and I felt that two sessions would be needed to cover the basic themes of my interviewing guide. The interviewees resided and worked across Finland, and all the interviews were conducted in Finnish.

I collected the data in the summer and autumn of 2020. All but one interview were conducted via Zoom – the exception was conducted by phone – and later transcribed by two companies providing transcription services. My method of gathering the data was an example of the remote qualitative method (Watson and Lupton, 2022). Unlike many scholars whose data collection required replanning because of the pandemic (Watson and Lupton, 2022), my choice of topic was enabled by it, and the plan was always to gather interviews remotely. All interviewees had comfortable access to a ‘screen’ (the one interviewed by phone also used videoconferencing in their work), and when clientele difficulties with accessing remote sessions were brought up in interviews this was framed in practical terms (e.g. organising childcare) rather than indicating any ‘digital divide’ (e.g. lack of equipment, poor connection or non-existent digital skills) (also Kolehmainen, 2022). This aligns with the high number of internet use and access in Finland: in 2020, 89% of households had an internet connection and 92% of the population had been online in the previous 3 months (Suomen virallinen tilasto [SVT], 2020).

During the data collection, the ‘first wave’ of the pandemic had hit Finland. This surely influenced the data-gathering process and the content of the interviews, from the urgency to collect data to the ways in which several interviewees were still feeling their way around the pivot to remote consultation. Several were only now learning to work remotely, and were still in the process of adjusting their practice to videoconferencing or phone call techniques. There was also some enthusiasm for the newfound pleasures of remote working. However, some of those who were most positive also evidently anticipated the pandemic being over soon. It is possible too that those having the most difficulty in shifting to work remotely either did not see my online call or did not want to be interviewed. I had no access to data about the interviewees’ clients. I asked about clients in the interviews, and interviewees referred to them constantly, but when they did so, it was always to give their professional interpretation. Furthermore, I have not attempted to compare different occupational groups, since many of my interviewees had several occupational qualifications. I will use only general occupational titles – such as psychotherapist or family counsellor – in order to protect my interviewees’ anonymity.

The first analytical strategy applied in processing the data was to employ a data-driven approach. My initial tentative analysis applied a light thematic approach to the transcripts. The procedure at this stage was to extract accounts and sort them into various categories. While the categories often overlapped, I assigned a single account to one category at that time, with in/visibility being one of the main themes. In fact, during this process, I became aware of the centrality of the theme of in/visibility in relation to the interviewees’ experiences. Professionals, clients and patients alike navigated their own visibility in very concrete ways, from switching their camera on and off to adjusting camera positions. There was a common concern of recognising how video calls allowed access to only limited visual information, but the visibility the screen technology provided was still valued. Some interviewees preferred traditional phone calls, but their accounts too involved vivid reflections on in/visibility.

In the second stage of the analysis, I applied a theory-driven analytical strategy to the data. I moved beyond a focus on the theme of in/visibility to thinking about not only the way in which screens are deployed in therapy practice but also how screens *themselves* act and operate (see Decuypere and Simons, 2016; Pols, 2011). This approach refers here both to the manifold operations and doings of the screen and to their particular component parts, for example, the texture of the digital screen, camera equipment, internet connection, and the platform or application in use. This take on screens accorded with that of Decuypere and Simons (2016): rather than viewing the digital as a general and neutral medium, I also sought rather to foreground the specificities and concrete operations of *the screen*, as the prototypical device associated with the digital. My data analysis method thus can best be described as thinking with theory (see St. Pierre and Jackson, 2014). Of course, as camera technology is inseparable from the screen in livestream settings, the interviewees’ talk about screens centres on various aspects of in/visibility, but the operations of screen can not be reduced onto that. I will next introduce these findings with the help of my rich empirical material. Extracts may have been slightly edited and shortened for readability.

## Visibility: On seeing and being seen

Even if a majority of my interviewees (but not all: for exceptions, see Kolehmainen, 2022) experienced remote work as burdensome, especially the limited access to visual information – staring at a screen and keeping still were among the things identified as exhausting – most still preferred video calls to phone calls because they provided some access to visual information:

*It’s about these subconscious issues, seeing facial expressions, and gestures, and such. But then again something is still missing, you get more out of live meetings, I don’t know what’s missing but I consider it second best, and a phone call is less suitable for [choosing] whether one dives deep or not.* (I20)

Like this crisis worker (I20), several of my interviewees developed a hierarchy, where in-person meetings are preferred but, in their absence, video calls are the next favourite medium since they provide access to visual information (see also Humer et al., 2020). This way, the screens were considered as *substitutes*, with videocalls seen as the second-best option when access to the in-person was restricted.

In particular, showing one’s face was valued. This aligns with how users of digital technology associate facial images with authenticity and trustworthiness (Pinch et al., 2022), whereas users without image are suspect – possibly fake, for instance. An interviewee who meets clients who have been violent, similarly connects showing one’s face with positive attributes such as responsibility:

*One really approaches a difficult issue with one’s own face and takes responsibility over one’s actions, it is also one thing that has to do with the fact that I would rather like to have [clients] on video or on the spot.* (I11)

Here, too, seeing is valued in a manner that renews the Western hierarchy of the senses, where sight is valued first and foremost and associated with the ‘truth’ (Synnott, 1997: 128–145). This interviewee interprets clients’ readiness to show their faces – no matter whether that takes place on screen or in a face-to-face meeting – as a sign of genuine willingness to change. From this point of view, the screens were seen as adding *value* to therapeutic encounters since revealing one’s face is read as a positive omen.

Users of videoconferencing technology usually do not see only one screen, but at least two: a video of the other user on a large screen, and a smaller screen showing the video from their own camera. Users are thus aware not only of the image of the other but also of their own onscreen appearance, which in the context of psychotherapy has been connected to detachment from the immediacy of *being there*: the smaller windows, especially, where one can see oneself are thought to be distracting (Isaacs Russell, 2015). Interviewees also reflected upon the experience of becoming self-consciously aware of one’s appearance onscreen:

*I believe that it’s sometimes even easier to work with the phone, since now, on Teams or on Zoom, you need to look at yourself all the time, the therapist and the client. It’s sometimes disturbing and a little distracting.* (I13)

This family counsellor notes that the therapist might also find seeing their own image all the time distracting: screen technology in a way ‘returns’ one’s gaze, as one cannot completely avoid seeing one’s own video. This duplicating of screens materialises in increasing self-awareness: the screen becomes a *mirror* that makes its presence in a therapy session clear.

The interviewees were able to make novel observations since screens are also *windows* onto new spaces and situations, through which a better understanding of the clients and patients can be attained. There is a difference between gazes relating to communication and those concerning observation (Pols, 2011: 459), although both might prove important in therapy work. This psychologist depicts a situation where she saw a child client having a tantrum at home:

*I was able to witness the thing this mother was worried about, like this child has absolutely outrageous tantrums [. . .] it was like super interesting that I had seen both what they embody with an adult they are a little unfamiliar with, then what it has been like there in the home environment.* (I27)

The interviewee recalls how video technology enabled observation of behaviour she had heard of but not previously witnessed, since this particular child’s behaviour differed according to environment. Here, technology confirmed and legitimised the concern of the child’s mother, since only the livestream revealed the tantrums at home.

The camera view was also purposefully used to ascertain whether the recipient of the call was alone. Thus, the screen served as evidential *proof*, documenting that no outsiders were in the call. In this way, the visual information that a professional was alone enhanced the psychic experience of trust. Another psychologist said some clients turned their videos off yet still wanted the interviewee to keep theirs on. At times, this was interpreted as the literal visualisation of mistrust:

*I too think that the fact that they see my picture is perhaps connected to issues of mistrust since they have been, like, a little suspicious regarding things like how to maintain connection and who hears and who sees, so I think it has been important to them to see I’m all alone.* (I7)

This extract importantly also reminds us that it was not only the professionals making observations. The clients were also using screen technology to observe. Moreover, this and other instances also provide examples of asymmetries between seeing and being seen. Being able to manage the camera view gives clients a chance to navigate power dynamics by trying to control and strike a balance between seeing and being seen.

## In/visibility: Not seeing or being seen

Like visibility, invisibility was a recurrent topic. In the interviews, not being able to see a client was often presented as a challenge in fulfilling professional goals. A psychotherapist articulately associates lack of visibility with a lack of quality in mental health care, explaining that a client might be audibly fine but visibly upset:

*I do want to see whether my client is crying, this is something I cannot necessarily hear on the phone. It has enormous significance if I proceed forward with leaps in some case and then the other person is like fraught if I cannot hear or register it well, that I would find quite horrid therapy.* (I38)

In particular, the interviewee is concerned about the impossibility of providing the experience of being held psychically. The concepts of therapeutic ‘holding’ (Winnicott, 1953) and ‘containing’ (Bion, 1984) are recurrent themes within psychodynamic theory and practice (Downing et al., 2021). While different from each other, the spatial metaphors of holding and containing are sometimes used interchangeably in order to refer to a therapist’s capacity to create a supportive emotional and/or physical space for the client (ibid.). In psychotherapy, holding and containing are thus prerequisites of a successful practice, but their establishment in a remote setting might prove a challenge, especially if the analyst ignores the particularities of the digital (Isaacs Russell, 2015: 14–15). Here, however, the interviewee is aware of those particularities and reflects upon the value of video calls, contrasting the phone option and related invisibility.

Visibility is, nevertheless, not a given. While videoconferencing platforms allow for visibility, human work is still required to position oneself on camera in order to be seen by participants. Camera angles are restricted, and the limited frame size requires people to be in the camera’s view if they want to be seen (Gan and DeSouza, 2022). Interviewees mention clients who have positioned themselves so as to remain partly out of view, or who – despite the video connection – refuse visibility:

*I thought the way in which people position themselves on camera was very telling, like there was one person I met, I don’t know how many times I met them before I saw their face. I mainly saw their forehead and the top of the head.* (I26)

A family counsellor describes the ‘telling’ way a particular client positions themself in front of the camera. This and other similar accounts demonstrate that clients can negotiate, refuse and reject visibility even on a video call by positioning themselves partly out of the camera’s eye. The client who only allowed his forehead to be seen did not lack the technical ability to make himself more visible, so the interviewee interpreted this as revealing. The interviewees read the screen view as revealing a *mental state* or indicating when a topic is sensitive: the screen thus provides information about the client.

However, the interviewees also explain that some clients opted to remain completely hidden from view. Here the screen is something like a *spotlight*, which might feel uncomfortable for clients who prefer to stay in the background. A psychotherapist points out that those who are used to staying invisible might opt for phone calls, allowing them to stay hidden, even where there are more practical reasons for the decision:

*I think that the kind of people who find it, who want to kind of stay hidden or who are used to stay invisible somehow, they did choose more often the option of having a phone connection, but then there might have been some partial reasons too [. . .]* (I21)

In some cases, the possibility of keeping out of view has enabled novel psychic processes to take place. In a very literal sense, the psychic states may become illustrated through the relations with screen, as a psychotherapist recalls:

*It’s not always the case that an adult perfectly masters this object constancy, and one of my clients, an individual client who wanted to shift to a phone contact, we realised this kind of, then we started to process it [. . .] Meaning that when they did not see me, whenever there was a silence, they checked whether I’m still there.* (I28)

This psychotherapist points out that the video – or lack thereof – makes psychic conditions visible. The interviewee associates the screen with *symptoms*: the webcam view does not visualise or represent symptoms; the symptoms emerge through their relations with screens. This also demonstrates how more-than-human agency entangles with mental well-being. I take the digital technology as becoming part of the enactment (see Mol, 2003) of both object constancy and its treatment in psychotherapy. Object constancy is a key concept in the field of developmental psychology and refers to the understanding that whether an object can be sensed has no effect on whether it continues to exist. Here the lack of visual access to the therapist, as a video call would have granted, triggers the client thus making the lack of object constancy known for the interviewed psychotherapist.

The challenges of reconfigurations of in/visibility were also consciously worked upon by some professionals. There were reflections on how to intentionally co-become with the screen in the data: some interviewees demonstrated adaptations to the screen, to the extent that technology itself becomes ‘invisible’ (Pols, 2011: 453–454) and a seamless part of the therapy-assemblage:

*I did notice at my therapy reception that I had to kind of learn, or to pay more attention as a therapist, to how one communicates with the client, like if the client is talking how does one actively listen via this laptop in a manner that the client gets the impression they are listened to.* (133)

This sex therapist, for instance, reflects upon evolving skills that enable communication with a client in a new way. Pols (2012) notes how telecare technologies prove their suitability for care in practice, because they can be categorised for fitting and unfitting technologies. Here the ‘fitting’ is a result of a process of learning how to listen. This is one example of intentional uses of the screen and how the screen needs to become a *match* to operate in a therapy or a counselling setting.

The becoming is not always intentional, though. There are accounts literally illustrating the co-becomings (differently) with the screen in the data. In the following extract, a psychotherapist describes becoming professionally different on- and offline:

*And that was the ‘kaboom’ about remote work, like okay, nothing is that different, it is just about the way I am. And I have noticed that I’m much more active concerning methods and activities compared to my office, as surprising it is. I don’t know which mode I fall into, we talk a lot on the head level but somehow I have been much braver, even with sensory exercises.* (I39)

She explains how the most significant moment for her view on remote therapy came when a client was triggered the very same day a shift to remote consultations took place, and she was able to help the client to ground. She recalls realising then that remote therapy is not worse than live, leading to her to experiment with sensory exercises. Thus, even if remote therapy limits sensory engagement, here, by contrast, the shift is addressed as mobilising sensory exercises.

I elaborated above some occasions of partial visibility, with interviewees recalling moments when clients were partially out of sight, but there were also more abrupt invisibilities. In particular, those interviewees who worked with couples recounted experiences of sudden disappearances from view, often when the relationship between the client couple was volatile:

*Well, these have happened if there’s a hot-tempered couple or a party. But then when [a client] disappears from the screen and like jumps out of the door it is different to a situation when a client leaves my office. It has a different meaning. I myself find it much more charged.* (I12)

Of course, sudden disappearances happen also for practical reasons, such as going to get water, but the professionals cannot know this without being told. Disappearances are also not limited to couples, but as this family counsellor (I12) explains, abrupt screen disappearances have a different feel to those happening in a shared space. She find them more charged. These co-becomings with the screen could be best described as *disentanglements*; refusals to connect with screen technology. Here the clients do not only keep out of the camera’s eye, they leave the setting altogether.

## The uses of screen

While the screen was used to appear visible or invisible, as I have demonstrated in the earlier sections of this article, screen technology was also used intentionally for various other purposes. For many professionals, this meant a possibility of engaging differently with their clients via digitally mediated technologies (Downing et al., 2021). Interviewees told how their clients have also purposefully used camera technology to show previously unseen aspects of their life worlds:

*Dogs have been introduced, cats have been introduced, one’s home has been introduced, one has wandered around the living room and the kitchen with a laptop, which I find absolutely wonderful. I think it is a sign of trust, if anything, and it is absolutely lovely that [they] want to [show] that side of their lives.* (I28)

This psychotherapist recalls being shown human and non-human family members and material surroundings. Disclosing one’s home and private life via webcam indeed happens usually when the participants are close (Pols, 2011: 465), in a similar vein, the interviewee interprets and experiences this as emotional closeness.

Interviewees also used visual information enabled by livestream to intentionally minimise the potentially alienating effects of the transition to remote sessions:

*And in the private sphere I then made use of my office room, in order to create a feeling of continuity for clients, so that they can see that I’m there in the same location, even if they cannot come there.* (I3)

*I have always come to sit here in my office, yes, and at the beginning I always signalled that here I am, there’s nobody in here, and then I shifted the view a little, like this is the familiar place and here I sit.* (I38)

It is known that some professionals seek to maintain stability and continuity by having their own background environment visible on the screen (Isaacs Russell, 2015: 15), like the family counsellor (I3) who describes using and showing the same venue used for in-person consultations to enhance feelings of continuity. Nevertheless, in my data this kind of webcam use overlaps with anticipated concerns about cybersecurity. By showing their office, a psychotherapist (I38) also offers the clients information about their surroundings, showing there are no other people present. Here the screen stands for the *continuity* of the patient/provider relationship.

Sometimes the possibility of working with multiple screen views – each client having their own equipment for accessing an appointment – was used to navigate a sensitive situation between clients. In this way, this possibility of having multiple screens becomes a part of the professional toolkit and enables de-escalation:

*So then I have had two clients, and I and they all had our own connections. They haven’t stayed in the same room. Partly because of COVID-related social distancing, or safety issues, but also because of tense relationships.* (I28*)*

*If the situation is inflamed and the clients find it difficult to stay in the same room, then using two screens remotely works just fine, at least temporarily.* (I23)

A psychotherapist (I28) explains that having more than two screen views (excluding the screens within screens, showing users their own camera view) could make possible some encounters that might not take place in-person at all, adding that the use of multiple screens also enabled observation of social distancing during the pandemic. Similarly, a family counsellor (I23) views the use of multiple screens as enabling sessions in situations where the clients’ mutual relationship is volatile. Here the screens work both as *digital masks* – similar to the actual medical masks worn during the COVID-19 pandemic, and the use of remote connection protects people from contamination (see Gourlay, 2022) and as *downers* to calm the situation – as the mutual relationships between clients is often volatile.

Interestingly, there were also mentions of screens other than those of the clients or the professionals. Indeed, the screens’ relations become networked in several ways, which for its part is telling of their centrality in contemporary modes of living. This family counsellor explains the procedures established to prevent any violations of privacy, such as the risk of clients having colleagues who can see their screens or share their electronic diary:

*And we decided that the title of that link is very neutral, like a meeting link, and there is no mention of couples therapy or suchlike. So if a client is on their office computer and someone else sees the title, they cannot get any information. And then we also did not add those meetings in their calendars, it was only an email link.* (I16)

Implicit Western assumptions of ‘one person/one device’ are predicated on the idea that one person controls access to for example, a phone (Pinch et al., 2022: 9), but the data make clear that this is a complicated assumption. As the last interviewee notes, many clients use devices and software owned by their employers, as well as accessing technology from work locations. One’s personal access to screens may in principle thus be easy, but more abstract and invisible networked connections such as synchronised electronic calendars mean heightened control over privacy.

## Wild screens

In the previous section, I pictured how humans use screen technology for various purposes, but digital technology can work both with and against the agency possessed by humans (Lupton, 2020). Screens themselves, with their unique forms of digital materiality (Sumartojo and Graves, 2021), shape the ways visual information can be accessed, searched for, and interpreted. With therapy at a distance, sitting before a screen constricts physical movement and eye contact is tricky (Markowitz et al., 2021: 242–243). A family counsellor (I3) describes the impossibility of direct eye contact via camera technology:

*If I understand these video calls correctly, like all the time we deal with delay, and if I look at the eye of a camera I still won’t see you in return in the eye of this camera, like that kind of a shared simultaneous gazing into one’s eyes is not possible.* (I3)

*Also, frozen expressions are something I have been thinking of. Since the aim is to maintain neutral facial expressions, but what if my screen would freeze too.* (I41)

Another family counsellor (I41) ponders the possibility of frozen screens, which exemplify a particular sort of suspended time – time that becomes stuck (Baraitser, 2017). The materiality of time indeed manifests itself through videoconferencing technology in several ways, and screens in turn twist chronological experiences of time and temporality. In both vignettes the screen marks a *cut* in all meanings of the word: physiological, psychological, affective. The camera technology produces actual and potential temporal cuts by rendering gazes apart, thus making real-time eye contact impossible, and by having the potential to pause livestream, generating a frozen screen. These kinds of disruptions to livestream pose challenges to the professional work conducted by therapists and counsellors.

Screens were discussed by interviewees as literally vibrant, too. It has been pointed out that even in wealthy countries, stable internet access is not guaranteed to the whole population but is mainly afforded by already privileged groups (Gonzales, 2016; Hargittai, 2021). However, there were hardly any descriptions of difficulties accessing suitable technology here (Kolehmainen, 2022). Consequently, vibrating screens were not discussed in relation to old equipment or low-speed connection, but in more practical terms:

*Some had a clear vision, like I want to go out for a run, and [then I had] to say that I find it too tiring to look at a vibrating picture, or if the connection cuts in and out, well then it’s better to have an ordinary call [. . .]* (I21)

A psychotherapist (I21) explains how looking at a vibrating picture feels like a challenge and how a livestream does not work if the client is moving. Even where difficulties in maintaining a stable connection were mentioned, they were not associated with unstable internet access. The unstable view is here a result of mechanical movement – a client running.

Earlier I noted occasions where clients disappeared from view and exited the session altogether. However, with two or more clients, the screen technology may be programmed into placing one client in the spotlight while hiding the other completely from view. A family counsellor and a psychotherapist recalls:

*Some of these computers have a screen that kind of gravitates in a way, I don’t know if it recognises movement or speech or something, and then the screen size changes, out of the blue the other disappears from view, and again. It has been a little disturbing every now and then.* (I10)

This is a reminder of the ‘wildness’ of technology – even if screens are used for certain purposes, their workings are also unpredictable for their users (Pols and Willems, 2011: 485), especially if they do not fully understand how the technology being used might be scripted. The gravitating of a screen here happens beyond the interviewee’s control, adding a new twist to the appointments.

Above, I introduced occasions where screens were experienced as barriers, physically and mentally. However, screen technology was also approached and experienced as a *bridge*, having the capacity to overcome potential and actual limitations in either remote or in-person consultations, thus synchronising the life-worlds of professionals and clients. A psychologist (I2) explains how some clients have taken advantage of being able to express themselves via the chat box, which is not possible when having meetings at an office:

*If it’s difficult to express oneself verbally for some particular reason, or there’s selective mutism or something else, in those cases I appeared as visible and audible as possible to them and they wrote their replies in the chat box and were able to express their thoughts perhaps in a more comprehensive way.* (I2)

However, being able to utilise the professional possibilities of screens often required human work, such as successful ‘taming’ of technology to make it work with, and not against, human (intentional) agency. A psychotherapist (I6) describes how screen technology is used to adjust the view, in particular the visible size of human bodies, in a manner that makes the distance feel appropriate.

*Like for instance distance becomes a concrete matter here, like you can ask a client do you want me to be like this or that [–] . We have brought it up even, like what could be a pleasant distance here, am I appearing too large on your screen now.* (I6)

*The basic thing in family therapy is to draw a family tree, which I find quite a nice and illustrative method, well that I have not even once done onscreen [. . .] This is such a thing that it would have been good to get some experience myself, to think how to have more possibilities, just like about the use of pics, like sharing photos.* (I5)

Another interviewee, a family counsellor (I5) regularly employs genograms – special family trees that visualise for instance intergenerational relationships and social patterns – in her work. While the importance of this professional method has subsided with the transition to remote consultations, the interview ponders the possibility of learning how to use screen technology for professional purposes. The screen is still approached as *potential*: a live texture that one could potentially learn to employ in encounters with clients. In other words, the agency of screens could work together with that of the interviewees and thereby generate new forms of agential capacities that are themselves useful in therapy and counselling work.

## Conclusions

In this article, I have demonstrated that digital screens are used for work, but they also operate in several ways in therapy and counselling settings, from allowing novel observations to purposeful visualisations, from providing psychological protection to offering physical shelter, moving from disturbing human-to-human communication to unpredicted operations. Screens come into being in manifold ways (Decuypere and Simons, 2016). In therapy and counselling settings, as my research shows, the screen can be *a window, a spotlight, a bridge, a digital mask*, and *a potential*, to name only a few of the possibilities. As Mol (2003) has vividly shown, in health care, a single object may appear to be more than one – and ‘the’ screen too refuses attempts to pigeonhole it as the one and only: it is and can be multiple, even during a single session. In health care, an understanding of the manifold operations of screen could support professionals in their work by allowing for more conscious, reflective, and controlled uses of digitally mediated technologies.

My analysis has illustrated how screens are material, haptic and ‘alive’. Drawing on Bennett’s (2010) theory of vitality of all matter, I propose that the screen can be conceptualised as ‘vibrant matter’. Its operations cannot be grasped by positioning it as a passive, flat man-made object (Bennett, 2010), but rather by acknowledging its vitality. Bennett remarks how even man-made items exceed their status as objects, having their own ‘thing-power’: they are vibrant things with an effect of their own. At the core of Bennett’s work lies the notion of distributed agency, where agency is not reduced to intentional human acts. Rather, she notes the distinctive capacities or efficacious powers of particular material configurations. From the perspective provided by the notion of distributed agency, objects such as screens do not possess thing-power on their own, but in relation with other elements of an assemblage, which may include humans (Bennett, 2004; Lupton, 2020). I thus argue that screens are not only ‘domesticated’ by humans in their efforts to provide (mental) care, but they co-constitute therapy sessions as part of different configurations. My conceptualisation of screens as vibrant recognises their own thing-power and pushes us forward in our understanding of them as part of healthcare settings. Even if I have not applied throughout neither the term ‘vibrant’ not the conceptions of vibrancy in my analysis, vital materialism features as an essential theoretical lens. I find the conceptualisation of screens as vibrant useful, as it *does* something: It invites us to see the digital screen as a material process; it recognises its multiplicity and draws attention to its agential capacities in specific contextualised settings as part of different configurations. This way, it complicates any dichotomous understanding between in-person and remote therapy.

Finally, I propose that screens can be seen as an essential part of more-than-human care in digitalised societies. The theory of more-than-human care departs from more traditional notions of care, where care is seen just as a human matter and where the categories of caring and cared-for play major roles (Puig de la Bellacasa, 2012, 2017). Aligning with work on more-than-human care (Puig de la Bellacasa, 2012, 2017), I argue that the position of screens in care does not rely on how humans ‘use’ them, nor can they only be understood to matter when they, for example, visualise a ‘care provider’, since humans and screens are constituted through their relationality. Further, acknowledging the agential capacities of all matter means recognising their potential in providing care (see also Méndez de la Brena, 2022); the screen thus also has capacities to act – or fail to act – as caring matter. However, as screens do not act on their own but possess thing-power that can be either constraining or enabling in relation with other elements of an assemblage (Lupton, 2019), there is variation how they foster mental health care. In this article, I have shown how, for instance, screens enhance care when they make certain mental states and conditions tangible, thus making room for intervention, or when they allow greater possibilities for self-expression or de-escalation; but they also might erode care by limiting the professionals’ possibilities for (literally) seeing what is going on or by making the use of therapeutic tools a challenge. This approach to mental health care thus enables us to consider how screens as part of therapy settings or counselling practices materialise – or fail to materialise – care.

## References

[bibr1-13634593241234491] Barad K (2012) On touching—the Inhuman that therefore I am. Differences 23(3): 206–223.

[bibr2-13634593241234491] Baraitser L (2017) Enduring Time. London: Bloomsbury Academic.

[bibr3-13634593241234491] Bennett J (2010) Vibrant Matter: A Political Ecology of Things. Durham, NC: Duke University Press.

[bibr4-13634593241234491] Bennett J (2004) The force of things: Steps toward an ecology of matter. Political Theory 32(3): 347–372.

[bibr5-13634593241234491] Bennett J (2020) Influx and Efflux: Writing Up With Walt Whitman. Durham, NC: Duke University Press.

[bibr6-13634593241234491] Bion WR (1984) Learning From Experience. London: Karnac Books.

[bibr7-13634593241234491] Cartwright L (1995) Screening the Body. Tracing Medicine’s Visual Culture. Minneapolis, MN: University of Minnesota Press.

[bibr8-13634593241234491] Coleman R (2008) The becoming of bodies. Feminist Media Studies 8(2): 163–179.

[bibr9-13634593241234491] Decuypere M Simons M (2016) What screens do: The role(s) of the screen in academic work. European Educational Research Journal 15(1): 132–151.

[bibr10-13634593241234491] Downing L Marriott H Lupton D (2021) ‘“Ninja” levels of focus’: Therapeutic holding environments and the affective atmospheres of telepsychology during the COVID-19 pandemic. Emotion Space and Society 40(100824). 10.1016/j.emospa.2021.100824 PMC9187329 35721520

[bibr11-13634593241234491] Gan Y DeSouza D (2022) Moving out of view: Managing participation and visibility in family video-mediated communication. Social Interaction. Video-Based Studies of Human Sociality 5(2). DOI: 10.7146/si.v5i2.125874

[bibr12-13634593241234491] Gonzales A (2016) The contemporary US digital divide: From initial access to technology maintenance. Information, Communication & Society 19(2): 234–248. DOI: 10.1080/1369118X.2015.1050438

[bibr13-13634593241234491] Gourlay L (2022) Digital masks: Screens, selves and symbolic hygiene in online higher education. Learning Media & Technology 47(3): 398–406.

[bibr14-13634593241234491] Hargittai E (2022) Connected in Isolation: Digital Privilege in Unsettled Times. Cambridge, MA: MIT Press.

[bibr15-13634593241234491] Humer E Stippl P Pieh C , et al. (2020) Experiences of psychotherapists with remote psychotherapy during the COVID-19 pandemic: Cross-sectional web-based survey study. Journal of Medical Internet Research 22(11). 10.2196/20246 PMC7704121 33151896

[bibr16-13634593241234491] Isaacs Russell G (2015) Screen Relations: The Limits of Computer-Mediated Psychoanalysis and Psychotherapy. Boca Raton, FL: Routledge.

[bibr17-13634593241234491] Kela (n.d.) Rehabilitative psychotherapy. Available at: https://www.kela.fi/rehabilitative-psychotherapy (accessed 19 January 2024).

[bibr18-13634593241234491] Kolehmainen M (2022) Intimate technology? Teletherapies in the era of COVID-19. In: Kolehmainen M Lahti A Lahad K (eds) Affective Intimacies. Manchester: Manchester University Press, pp.63–80.

[bibr19-13634593241234491] Kuhn A (2009) *Screen* and screen theorizing today. Screen 50(1): 1–12.

[bibr20-13634593241234491] Lupton D (2019) Toward a more-than-human analysis of digital health: Inspirations from feminist new materialism. Qualitative Health Research 29(14): 1998–2009. 30964392 10.1177/1049732319833368

[bibr21-13634593241234491] Lupton D (2020) Vital Materialism and the Thing-Power of Lively Digital. Data. In: Leahy D Fitzpatrick K Wright J (eds) Social Theory and Health Education: Forging New Insights in Research. 1st edition [Online]. Milton: Routledge.

[bibr22-13634593241234491] Markowitz JC Milrod B Heckman TG , et al. (2021) Psychotherapy at a distance. American Journal of Psychiatry 178(3): 240–246. 32972202 10.1176/appi.ajp.2020.20050557

[bibr23-13634593241234491] McCarthy A (2001) Ambient Television: Visual Culture and Public Space. Durham, NC: Duke University Press.

[bibr24-13634593241234491] Méndez de la Brena E (2022) Caring matter: A love story of queer intimacies between (her) body and object (her cigarette). In: Kolehmainen M Lahti A Lahad K (eds) Affective Intimacies. Manchester: Manchester University Press, pp.27–44.

[bibr25-13634593241234491] Mol A (2003) The Body Multiple: Ontology in Medical Practice. Durham, NC: Duke University Press.

[bibr26-13634593241234491] Mol A (2008) The logic of care: Health and the problem of patient choice. London: Routledge.

[bibr27-13634593241234491] Pinch A Birnholtz J Rawat S , et al. (2022) Someone else is behind the screen’: Visibility, privacy, and trust on geosocial networking apps in India. Social Media + Society 8(3). DOI: 10.1177/20563051221126076

[bibr28-13634593241234491] Pink S Ardèvol E Lanzeni D (eds) (2016) Digital Materialities: Design and Anthropology. London: Bloomsbury.

[bibr29-13634593241234491] Pols J (2011) Wonderful Webcams: About Active Gazes and Invisible Technologies. Science Technology & Human Values 36(4): 451–473.

[bibr30-13634593241234491] Pols J (2012) Care at a Distance: On the Closeness of Technology [Online]. Amsterdam: Amsterdam University Press.

[bibr31-13634593241234491] Pols J Willems D (2011) Innovation and evaluation: Taming and unleashing telecare technology. Sociology of Health & Illness 33(3): 484–498. 21241338 10.1111/j.1467-9566.2010.01293.x

[bibr32-13634593241234491] Puig de la Bellacasa M (2012) Nothing comes without its world’: Thinking with care. Sociological Review 60(2): 197–216.

[bibr33-13634593241234491] Puig de la Bellacasa M (2017) The Matters of Care. Minneapolis, MN: University of Minnesota Press.

[bibr34-13634593241234491] Shulman G (2021) The screen object. Journal of Child Psychotherapy 47(2): 269–295. 10.1080/0075417X.2021.1954977 PMC7612633 35444352

[bibr35-13634593241234491] Sobchack VC (2004) Carnal thoughts Embodiment and Moving Image Culture [Online]. Berkeley: University of California Press.

[bibr36-13634593241234491] St. Pierre EA Jackson AY (2014) Qualitative data analysis after coding. Qualitative Inquiry 20: 715–719.

[bibr37-13634593241234491] Sumartojo S Graves M (2021) Feeling through the screen: Memory sites, affective entanglements, and digital materialities. Social & Cultural Geography 22(2): 231–249.

[bibr38-13634593241234491] Suomen virallinen tilasto (SVT) (2020) Väestön tieto- ja viestintätekniikan käyttö [verkkojulkaisu]. ISSN=2341-8699. Liitetaulukko 11. Internetin käyttö ja käytön useus 2020, %-osuus väestöstä. Helsinki: Tilastokeskus (accessed 16 October 2023).

[bibr39-13634593241234491] Synnott A (1997) The Body Social: Symbolism, Self and Society. London: Routledge.

[bibr40-13634593241234491] Trondsen MV Tjora A Broom A , et al. (2018) The symbolic affordances of a video-mediated gaze in emergency psychiatry. Social Science & Medicine 197: 87–94. 29222999 10.1016/j.socscimed.2017.11.056

[bibr41-13634593241234491] Watson A Lupton D (2022) Remote fieldwork in homes during the COVID-19 pandemic: Video-call ethnography and map drawing methods. International Journal of Qualitative Methods 21: 1–12. 10.1177/16094069221078376 PMC8922046 35309898

[bibr42-13634593241234491] Williams N (2021) Working through COVID-19: ‘Zoom’ gloom and ‘Zoom’ fatigue. Occupational Medicine 71(3): kqab041.

[bibr43-13634593241234491] Winnicott D (1953) Transitional objects and transitional phenomena; a study of the first not-me possession. International Journal of Psychoanalysis 34(2): 89–97. 13061115

